# Immune Checkpoint Inhibitor-Related Cerebellar Toxicity: Clinical Features and Comparison with Paraneoplastic Cerebellar Ataxia

**DOI:** 10.1007/s12311-024-01727-5

**Published:** 2024-08-17

**Authors:** Marta Dentoni, Irene Florean, Antonio Farina, Bastien Joubert, Le-Duy Do, Jérôme Honnorat, Valentina Damato, Martina Fabris, Gian Luigi Gigli, Mariarosaria Valente, Alberto Vogrig

**Affiliations:** 1 Clinical Neurology, Department of Medicine (DMED), University of Udine, Udine, Italy; 2 Clinical Neurology, Department of Head-Neck and Neuroscience, Azienda Sanitaria Universitaria Friuli Centrale (ASUFC), Udine, Italy; 3 French Reference Centre for Paraneoplastic Neurological Syndromes and Autoimmune Encephalitis, Hospices Civils de Lyon, Lyon, France; 4 MeLiS - UCBL-CNRS UMR 5284 - INSERM U1314, Université Claude Bernard Lyon 1, Lyon, France; 5 Department of Neurosciences, Drugs and Child Health, University of Florence, Firenze, Italy; 6 Laboratory of Immunopathology, Institute of Clinical Pathology, Department of Laboratory Medicine, University Hospital of Udine, Udine, Italy; 7 Institute of Clinical Pathology, Department of Medicine (DMED), University of Udine, Udine, Italy

**Keywords:** Neurological adverse events, Immune-related adverse events, Neurological toxicities, Autoimmune encephalitis, Paraneoplastic neurological syndromes

## Abstract

Immune checkpoint inhibitors (ICIs) have revolutionized cancer therapy, and the association with immune-related adverse events (irAEs) is well-established. However, cerebellar irAEs are poorly defined and their relationship with paraneoplastic disorders remains unclear. Our aim was (i) to characterize cerebellar irAE; (ii) to compare it with paraneoplastic cerebellar ataxia (PCA). We performed a multicenter, retrospective, cohort study of patients developing new-onset, immune-mediated, isolated/predominant cerebellar dysfunction after ICI administration. In addition, a systematic review following PRISMA guidelines was performed. Cerebellar irAE cases were compared with a consecutive cohort of patients with PCA. Overall, 35 patients were included, of whom 12 were original cases (males: 25/35 (71%), median age: 65 [range: 20–82]). The most frequent tumor was non-small cell lung cancer (12/35, 34%). Anti-PD1 were adopted in 19/35 (54%). Symptoms developed at a median of 11 weeks after ICI onset. Neuronal antibodies were detected in 15/31 patients tested (48%). Cerebrospinal fluid was inflammatory in 25/30 (83%). Magnetic resonance imaging showed cerebellar hyperintensities in 8/35 (23%). Immunotherapy was applied in 33/35 cases (94%), and most patients improved with residual disability (16/35, 46%). When compared with a series of PCA (*n* = 15), the cerebellar irAE group was significantly more associated with male sex, lung cancer (rather than gynecological/breast cancers), isolated ataxia, and a better outcome. We provide a detailed characterization of cerebellar irAE. Compared to PCA, differences exist in terms of tumor association, clinical features, and outcome. Clinical presentation-antibody-tumor triad in the ICI group only partially reflects the associations described in paraneoplastic disorders.

**Supplementary Information:**

The online version contains supplementary material available at 10.1007/s12311-024-01727-5.

## Introduction

Since their introduction in 2011, immune checkpoint-inhibitors (ICIs) have revolutionized the field of cancer immunotherapy [1]. T cells action is modulated by several costimulatory and coinhibitory receptors. Among them, PD-1 (programmed death 1), PD-L1 (PD-1 ligand), and CTLA-4 (cytotoxic T lymphocyte antigen 4) play a role as negative regulators: the activation of these receptors therefore promotes self-tolerance and prevents autoimmunity, but it can be exploited by cancer cells to evade the immune system [1]. ICIs unbalance the system towards T cell activation, thus countering the immune suppression in the tumor microenvironment and promoting appropriate anticancer response. However, treatment with ICIs may as well induce immune-related adverse events (irAEs) [2]. Among autoimmune complications, neurological irAEs involve approximately 1–12% of the patients treated with ICIs [3, 4] and they seem to preferentially affect the peripheral nervous system over the central nervous system (CNS), twice or even three times as commonly [5, 6].

Therefore, our knowledge on irAEs affecting the CNS remains limited to case reports and small case series [7, 8] and, within this group, cerebellar irAEs are among those with less available data on clinical course, immunological associations, and outcome [6].

In addition, the relationship between cerebellar irAEs and their naturally occurring paraneoplastic counterpart (paraneoplastic cerebellar ataxia, PCA) remains unclear [9], despite the fact that this is an interesting paradigm for which also an animal model exists, suggesting a defect in coinhibitory pathways being involved in “spontaneous” paraneoplastic neurological syndromes (PNS) [10].

Herein, we provide a characterization of cerebellar irAEs by means of a multicenter, retrospective, cohort study along with a systematic literature review. The cases of cerebellar irAEs were also compared to a consecutive original series of patients with PCA.

## Materials and Methods

### Patient Selection

The present study is a multicentric, retrospective, cohort study of patients who developed new-onset, immune-mediated, isolated or predominant cerebellar dysfunction within 12 months from the last ICI administration [5] between January 1, 2017, to October 17, 2023. Patients were included from two Italian hospitals, both tertiary referral centers, each covering a population in the range of approximately one million people for the diagnosis and treatment of patients with PNS, autoimmune encephalitis (AE), and neurological irAEs related to cancer immunotherapy (*Azienda Sanitaria Universitaria Friuli Centrale*, Udine, Italy; *Azienda Ospedaliero-Universitaria Careggi*, Firenze, Italy) and the French National Reference Center for AE and PNS (*Centre de Référence des Syndromes Neurologiques Paranéoplasiques et Encéphalites Auto-immunes*, Lyon, France), which provides countrywide antibody (Ab) testing and clinical care for suspected cases of autoimmune neurologic syndromes (total population covered of approximately 65 million people).

For the purpose of this study, we searched the database of the three centers for patients who developed signs and symptoms suggestive of cerebellar involvement as the core neurological manifestation, without any relevant extra-cerebellar involvement and in whom no alternative (neoplastic, infectious, metabolic, genetic, or structural) cause was found other than the toxicity due to ICIs.

Symptoms of cerebellar dysfunction were classified as dysarthria or scanning speech, oculomotor cerebellar deficit (namely nystagmus, ocular dysmetria and saccadic intrusions), gait ataxia, truncal ataxia, limb ataxia or dysmetria, and dizziness or vertigo [11]. We termed “isolated cerebellar ataxia” the isolated presence of gait and/or limb and/or trunk ataxia, and we defined “pancerebellar syndrome” the concomitant presence of ataxia, dysarthria, and ocular involvement.

ICIs considered were ipilimumab and tremelimumab, targeting CTLA-4; nivolumab, pembrolizumab and toripalimab, targeting PD-1; atezolizumab, avelumab, and durvalumab, targeting PD-L1; relatlimab, targeting Lymphocyte-Activation Gene-3 (LAG-3).

Clinical and ancillary data, including results of cerebrospinal fluid (CSF) analysis and magnetic resonance imaging (MRI) of the brain, were extracted by retrospective medical records review.

A concentration > 50 mg/dL was considered the pathologic threshold for protein content, while a cell count ≥ 5 per mm^3^ was considered pathological for cells in the CSF [12]. Type II and type III oligoclonal bands (OCBs) were considered when defining CSF as inflammatory [13], but the presence of other patterns (e.g. “mirror pattern” or type IV) was also recorded. The samples (serum and CSF) of the patients were systematically tested with indirect immunofluorescence on rat brain sections as initial assessment, followed by a second confirmatory test—dot blot analysis on recombinant proteins (Euroimmun, Lübeck, Germany) or cell-based assays (in-house techniques)—for the presence of neuronal Abs. Neurological outcomes were assessed according to the following categories: return to pre-ICI condition, improvement with residual disability, absence of improvement, or worsening. Oncological outcomes consisted in complete/partial response, absence of progression, or tumor progression/recurrence.

### Literature Review

A systematic review of the literature was conducted following the Preferred Reporting Items for Systematic Reviews and Meta-Analyses (PRISMA) guidelines. A comprehensive search in MEDLINE (PubMed) was performed on October 17th, 2023, using the following search string: “(cerebellitis OR pancerebellitis OR cerebellar encephalitis OR “cerebellar”) AND (ipilimumab OR nivolumab OR pembrolizumab OR atezolizumab OR avelumab OR durvalumab OR cemiplimab OR immune checkpoint inhibitor)”. The same inclusion criteria employed for patient selection were adopted for the literature review. Consequently, for a patient to be included in the study, clinical information had to be assessable at an individual patient level and the full article had to be available for review (abstract-only papers were excluded).

Each article was screened for patient sex and age, clinical features, and oncological data including tumor type and ICI adopted. When case reports generically described symptoms as “ataxia”, this was interpreted as ataxia of gait. Time lag between onset of ICI therapy and onset of neurological symptoms was also recorded; few articles provided the number of therapy cycles only, not describing the exact number of weeks. In such cases approximations were made according to Food and Drug administration (FDA) and European Medical Agency (EMA) safety data on treatment protocols.

Relevant findings on lumbar puncture were also collected (CSF protein level, cellularity, tumor cells, OCBs). Neuronal Ab presence and type, brain MRI features (cerebellar atrophy, edema, hyperintensities, or others), immune modulating therapy applied (corticosteroids, intravenous immunoglobulins, plasma exchange, rituximab, or others), neurological and oncological outcome, and if specified, cause of death were also recorded.

**Fig. 1:**
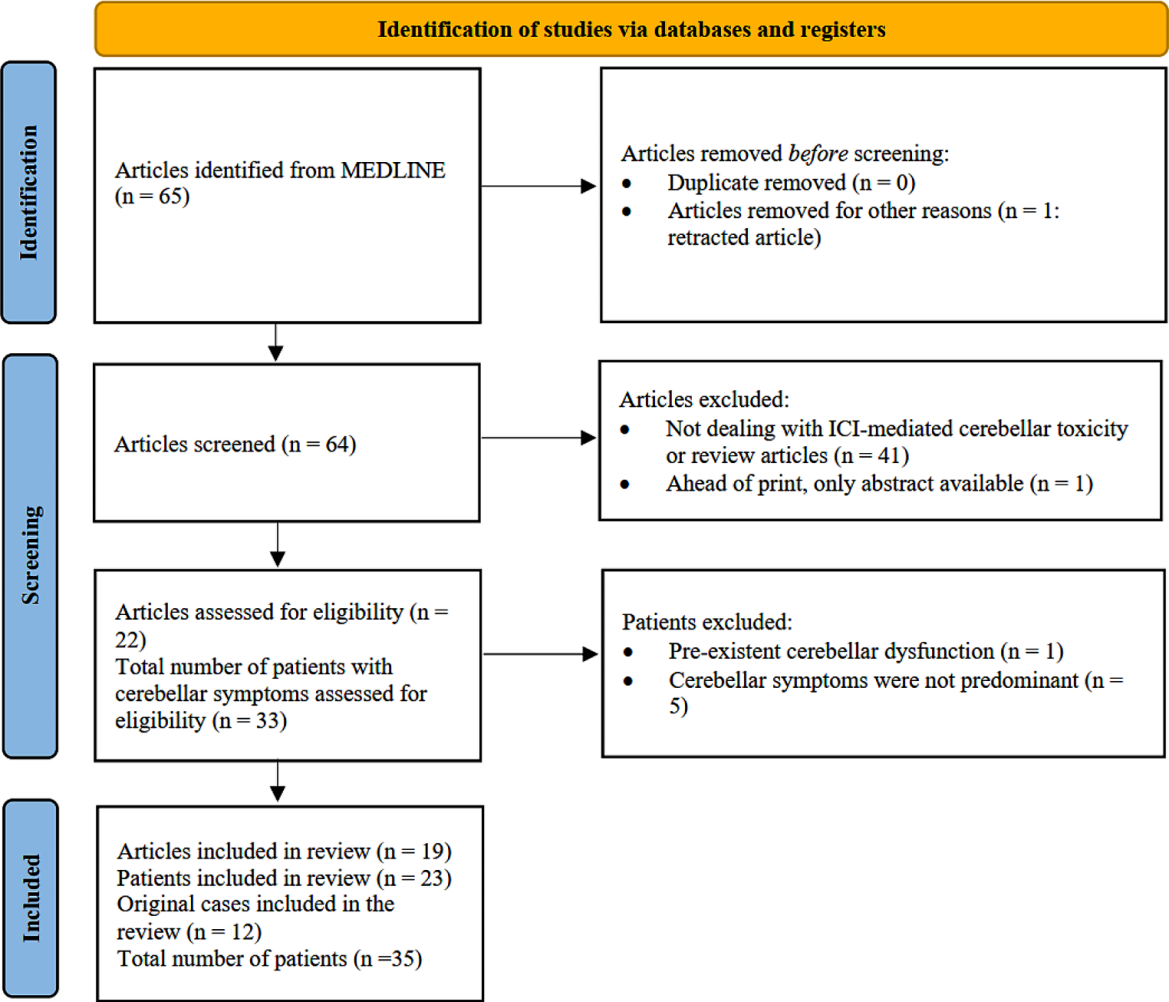
Preferred Reporting Items for Systematic Reviews and Meta Analyses (PRISMA) flowchart

M.D. and I.F. performed the initial selection, review, and extraction of patients’ clinical and paraclinical data, while A.V. supervised the entire systematic review process. The PRISMA flow diagram is shown in Fig. 1.

### Comparison to Paraneoplastic Cerebellar Syndromes

Patients with cerebellar irAEs were compared with a consecutive cohort of patients with PCA, unrelated to ICI exposure, diagnosed at the Udine University Hospital (from January 1, 2017, to October 17, 2023). Patients included in this control cohort had (i) a diagnosis of “possible”, “probable”, or “definite” PNS according to the PNS-care score [14]; (ii) an isolated or predominant cerebellar syndrome; (iii) exclusion of alternative etiologies. Collected data included sex, age, and oncological information (tumor type, presence/absence of antecedent tumors, previous oncological therapies, and metastases at tumor diagnosis). Symptoms of cerebellar dysfunction and paraclinical tests were classified using the same definitions as the cerebellar irAE group. Time lag between onset of neurological symptoms and histological tumor diagnosis was also recorded and approximated in weeks.

### Statistical Analysis

Descriptive analysis is presented as frequencies and percentages for categorical variables and as median and range for continuous variables. Comparison between proportions was performed by means of χ^2^ and Fisher’s exact test. GraphPad QuickCalcs Web site was used for *P value* calculation (https://www.graphpad.com/quickcalcs/contingency1/, accessed February 2024). P values < 0.05 (two-tailed) were considered statistically significant.

## Results

### Clinical Vignettes of ICI-Related Cerebellar Toxicity

#### Patient 1

A 71-year-old woman with Human Papilloma Virus (HPV)-related anal squamous cell carcinoma received atezolizumab. Two months after anti-PD-L1 initiation, she developed a grade (G) 2 ankle arthritis, followed by G3 hyponatremia 7 months after the onset of treatment, both diagnosed as irAEs. In addition, dizziness and postural instability appeared during active atezolizumab treatment, 14 months after its initiation. Symptoms progressed in the subsequent 2 months, as she developed dysarthria, gait ataxia, retropulsion, and upper limbs dysmetria. Lumbar puncture (LP) revealed normal protein content and cellularity, but CSF-restricted OCBs were detected. Neuronal Abs were negative. Contrast-enhanced brain MRI did not reveal any cerebellar abnormality. Lower limbs nerve conduction studies were unremarkable. High-dose corticosteroids (CS) were administered (8-day-course of IV methylprednisolone 500 mg/die, followed by oral taper), and a significant improvement of gait and limb ataxia was noticed. However, during CS oral taper (50 mg/die) she experienced neurological worsening, including limb ataxia, dysarthria, and dysphagia. From an oncological standpoint, the patient was disease-free at latest follow up, over three years after ICI withdrawal. However, her neurological status kept worsening: at a 4-year follow-up, she was wheelchair-bound and had developed frank truncal ataxia (Video 1); both upper and lower limb ataxia appeared; dysarthria became severe and ocular dysmetria added up to the clinical picture.

#### Patient 2

A 60-year-old man with lung adenocarcinoma received pembrolizumab, with concomitant platinum and pemetrexed chemotherapy. Five weeks after ICI initiation, pancerebellar symptoms appeared subacutely, including dysarthria/scanning speech, oculomotor disturbances, gait, trunk, and limb ataxia. LP revealed mildly increased protein content (53 mg/dL) and pleocytosis (19 cells/mm^3^). Anti-Yo Abs were detected. Brain MRI was unremarkable. Pembrolizumab was withdrawn and the patient was treated with CS and intravenous immunoglobulins (IVIg), with no improvement. Oncological disease progression was observed, and the patient eventually died (follow-up time: 4 months).

#### Patient 3

A 52-year-old woman with ovarian clear cell carcinoma was treated with pembrolizumab; concomitant bevacizumab and doxorubicin therapy was administered. Three weeks after ICI initiation, she developed dysarthria and trunk, gait and limb ataxia. LP revealed increased protein content (59 mg/dL) and pleocytosis (64 cells/mm^3^). Anti-Yo positivity was identified, while MRI was normal. Pembrolizumab was withdrawn, while CS, IVIg and rituximab (RTX) were administered, with no effect on her neurological status. The underlying cancer progressed, and she eventually died (follow-up time: 17 months).

#### Patient 4

A 66-year-old woman was treated with a combination of nivolumab and relatlimab for a retroperitoneal leiomyosarcoma. Eight weeks after starting ICI therapy, she developed gait and limb ataxia. LP revealed increased protein content (368 mg/dL), pleocytosis (261 cells/mm^3^), and type II OCBs. Neuronal Ab and microbiological testing as well as brain MRI were negative. ICI combination therapy was withdrawn, and CS and IVIg were administered, leading to symptoms improvement despite residual disability. Oncological disease recurred (follow-up time: 8 months).

#### Patient 5

A 53-year-old woman with a history of small cell lung cancer (SCLC) was treated with atezolizumab and concomitant platinum-etoposide. Eleven weeks from the initiation of atezolizumab, she developed dizziness, dysarthria, oculomotor cerebellar deficits and limb ataxia. In addition, she became confused and dysphagic. CSF testing revealed normal proteins, pleocytosis (21 cells/mm^3^), and OCBs positivity. Anti-TRIM9 positivity was detected. MRI showed bilateral III and right VII-VIII cranial nerve contrast enhancement, while no cerebellar abnormality was detected. Despite ICI interruption and treatment with CS, IVIg and tofacitinib (Janus Kinase (JAK) inhibitor), no improvement of cerebellar symptoms was noticed. However, no tumor progression was detected either at oncological follow-up (follow-up time: 5 months).

#### Patient 6

A 79-year-old man with melanoma was treated with 4 cycles of a combination ICIs (nivolumab and ipilimulab), followed by nivolumab alone. During active ICI treatment (eighty-two weeks after ICIs were initiated), gait and limb ataxia were noticed. In addition to ICI-mediated cerebellar toxicity, other irAEs developed, including vitiligo and thyroiditis. LP revealed increased CSF proteins (100 mg/dL), but neither pleocytosis nor OCBs presence. No neuronal Abs positivity was detected, and brain MRI was negative. ICI interruption and CS treatment led to symptoms improvement with residual disability (follow-up: 15 months). Oncological outcome was not available.

#### Patient 7

A 75-year-old man with a history of SCLC was treated with atezolizumab and platinum-etoposide concomitantly with the first 4 cycles of ICI. During active atezolizumab treatment (44 weeks after its initiation), a pancerebellar syndrome developed, including dysarthria, oculomotor cerebellar abnormalities, as well as gait, truncal and limb ataxia. CSF proteins and cell count were normal, while CSF-specific OCBs were detected. Atypical Ab staining was initially observed, and anti-DACH1 positivity was later identified; MRI showed cerebellar atrophy and cerebellar hyperintensities. ICI was withdrawn, and CS and IVIg were started, without any significant improvement (immunotherapy led to mild dysarthria amelioration, but the benefit was transient). From an oncological point of view, tumor progression was noticed (follow-up time: 14 months).

#### Patient 8

A 68-year-old man with stage IV lung adenocarcinoma received nivolumab, after several other anticancer treatment attempts (stereotactic radiotherapy, cisplatin, 5-fluoro-uracyl, and cetuximab). During active nivolumab treatment (25 weeks after its initiation), gait and trunk ataxia, and oculomotor cerebellar deficits developed. LP revealed normal CSF proteins and cell count, and OCBs negativity. Ab screening was negative, while MRI revealed cerebellar hyperintensities involving dentate nuclei bilaterally. Nivolumab was withdrawn, and CS were administered; from a neurological standpoint, he improved with residual disability, and no cancer progression was demonstrated but the patient was lost to follow-up.

#### Patients 9–12

Four cases of cerebellar irAEs were seen during the study period and previously described by our group in a paper focused on CNS adverse events of ICI [7]. These cases were not described at the individual patient level in the initial publication, but rather as a whole. We therefore retrieved clinical and paraclinical data of the individual patients, obtaining a total of 12 original cases.

### Clinical Features of Cerebellar irAEs in a Cohort of 35 Cases

Overall, 19 articles with 23 individual patients were included in the systematic review, to which our 12 original cases of cerebellar irAEs were added. Relevant characteristics of patients with ICI-mediated cerebellar toxicity are shown in Table 1.

**Table 1:** Characteristics of patients with cerebellar irAEs

Reference	Sex, age	ICI	Underlying tumor	time lag onset of therapy-neurological symptoms (weeks)	Cerebellar symptoms	Abs	MRI/CSF (increased protein content, pleocytosis, OCBs (type))	Treatment	Neurological outcome
Original case 1	W, 71	atezolizumab	Anal SCC	60	Pancerebellar	-	-/--+(II)	CS	3
Original case 2	M, 60	pembrolizumab	NSCLC	5	Pancerebellar	Yo	-/++NA	CS, IVIg	2
Original case 3	W, 52	pembrolizumab	Ovarian CCC	3	Ataxia + dysarthria	Yo	-/++NA	CS, IVIg, RTX	2
Original case 4	W, 66	nivolumab + relatlimab	Retroperitoneal leiomyosarcoma	8	Isolated ataxia	-	-/+++(II)	CS, IVIg	1
Original case 5	W, 53	atezolizumab	SCLC	11	Pancerebellar	TRIM9	-/-++(NA)	CS, IVIg, tofacitinib	2
Original case 6	M, 79	nivolumab + ipilimulab §	Melanoma	82	Isolated ataxia	-	-/+--	CS	1
Original case 7	M, 75	atezolizumab	SCLC	44	Pancerebellar	DACH1	Cerebellar atrophy and hyperintensities/--+(II)	CS, IVIg	2
Original case 8	M, 68	nivolumab	NSCLC	25	Ataxia + ocular	**-**	Cerebellar hyperintensities/---	CS	1
Original case 9	M, 32	nivolumab	Hodgkin lymphoma	10	NC	UNCA	-/++NA	CS	0
Original case 10	M, 72	nivolumab	NSCLC	13	Ataxia + ocular	UNCA	Cerebellar atrophy/+++(NA)	CS, IVIg	1
Original case 11	M, 70	atezolizumab	SCLC	7	NC	Hu	-/+++(NA)	CS, IVIg	2
Original case 12	M, 62	durvalumab + ipilimumab	Bladder carcinoma	69	Isolated ataxia	-	-/+-NA	CS	0
Kao et al., 2017 [32]	M, NA	pembrolizumab	NSCLC	30	Ataxia + dysarthria	NA	-/NA	none	1
Kawamura et al., 2017 [33]	W, 54	nivolumab	NSCLC	2	Ataxia + ocular	-	-/++NA	CS	0
Naito et al., 2018 [34]	M, 57	nivolumab + ipilimumab	SCLC	8	Pancerebellar	-	Cerebellar edema and hyperintensities/++NA	CS, PE, RTX	1
Vitt et al., 2018 [35]	M, 70	pembrolizumab	SCC of the neck	30	Pancerebellar	-	Cerebellar hyperintensities/+++(II)	CS	1
Zurko et al., 2018 [36]	M, 20	nivolumab	Hodgkin lymphoma	6	Isolated ataxia	NA	Cerebellar edema and hyperintensities/++NA	CS	1
Saikawa et al., 2019 [37]	M, 71	pembrolizumab	NSCLC	6	Ataxia + dysarthria	NA	-/++NA	CS	0
Tan et al., 2019 [38]	M, 66	atezolizumab	NSCLC	17	Isolated ataxia	-	-/--+(IV)	CS	1
Iyer et al., 2020 [39]	M, 37	nivolumab + ipilimumab	Head and neck SCC	6	Ataxia + dysarthria	Zic4	-/NA	CS, IVIg, PE, RTX	1
Monteiro et al., 2020 [40]	M, 82	pembrolizumab	MCC	39	Ataxia + dysarthria	-	-/+-+(IV)	CS, IVIg	3
Sanchis-Borja et al., 2020 [41]	M, 63	pembrolizumab	NSCLC	12	NC	GFAP	-/++NA	CS	0
Sanchis-Borja et al., 2020 [41]	M, 70	nivolumab	NSCLC	18	Isolated ataxia	-	-/+-NA	CS	0
Sanchis-Borja et al., 2020 [41]	M, 77	nivolumab	NSCLC	6	Ataxia + dysarthria	-	Cerebellar hyperintensities/+-NA	CS	0
Hardwick et al., 2021 [42]	M, 63	ipilimumab	SCLC	37	Pancerebellar	Yo, VGCC	-/+-+(IV)	CS, infliximab	3
Schmidt et al., 2021 [43]	M, 47	nivolumab + ipilimumab	Melanoma	5	Isolated ataxia	NA	-/++NA	CS	1
Segal et al., 2021 [44]	W, 50	nivolumab	Renal CCC	6	Ataxia + ocular	PCA-2 (MAP1B)	Cerebellar hyperintensities/--+(II)	CS	1
Zhou et al., 2022 [23]	W, 63	toripalimab	Melanoma	0.1	Pancerebellar	GAD65	-/+-NA	CS, IVIg, PE	1
Dinoto et al., 2022 [45]	W, 67	avelumab	MCC	3	Isolated ataxia	NF-L, NF-H, alpha-internexin	-/++NA	CS, IVIg	1
Koch et al., 2022 [46]	M, 52	pembrolizumab	NSCLC	34	Isolated ataxia	-	Cerebellar hyperintensities/NA-NA	CS	1
Valencia-Sanchez et al., 2022 [47]	M, 69	atezolizumab	SCLC	11	Isolated ataxia	Hu	-/---	CS, IVIg, cyclophosphamide	1
Valencia-Sanchez et al., 2022 [47]	W, 64	atezolizumab	SCLC	53	Isolated ataxia	-	-/--NA	CS	0
Valencia-Sanchez et al., 2022 [47]	M, 71	pembrolizumab	NSCLC	31	Isolated ataxia	-	Cerebellar atrophy/NA	none	2
Valencia-Sanchez et al., 2022 [47]	W, 70	nivolumab	SCLC	NA	Isolated ataxia	Amphiphysin, P/Q VGCC (S)	-/NA	CS, IVIg, PE	2
Chen et al., 2023 [48]	M, 46	atezolizumab	SCLC	3 cycles + 2 weeks	Pancerebellar	-	Cerebellar atrophy/--NA	CS	0

*Abbreviations* Ab, antibody; CCC, clear cell carcinoma; CS, corticosteroids; CSF, cerebrospinal fluid; GFAP, glial fibrillary acidic protein; ICI, immune-checkpoint inhibitor; irAE, immune-related adverse event; IVIg, intravenous immunoglobulins; M, man; MCC, Merkel cell carcinoma; MRI, magnetic resonance imaging; NA, not available; NC, not characterized; NF-H, neurofilament-heavy chain; NF-L, neurofilament-light chain; NSCLC, non-small cell lung cancer; OCB, oligoclonal band; PE, plasma exchange; RTX, rituximab; SCC, squamous cell carcinoma; SCLC, small cell lung cancer; UNCA, antibodies binding to unclassified antigens; VGCC, voltage-gated calcium channels; W, woman; +, present; -, normal

Neurological outcome: 0 = return to pre-ICI condition; 1 = improved with residual disability; 2 = no improvement; 3 = worsening

§ combination therapy for 4 cycles, then nivolumab alone

Among a total of 35 cases of cerebellar toxicity following ICI therapy identified, 25/35 (71%) were males, while 10/35 (29%) females. The median age (available in 34/35 cases) was 65 years (age range: 20–82). The most frequent tumor was non-small cell lung cancer (NSCLC), which was present in 12/35 (34%) patients, followed by SCLC (9/35, 26%), melanoma (3/35, 9%), Hodgkin lymphoma (2/35, 6%), head and neck squamous cell carcinoma (HNSCC; 2/35, 6%), Merkel cell carcinoma (MCC; 2/35, 6%), bladder carcinoma (1/35, 3%), renal cell carcinoma (RCC; 1/35, 3%), HPV-related anal squamous cell carcinoma (1/35, 3%), ovarian clear cell carcinoma (1/35, 3%), and retroperitoneal leiomyosarcoma (1/35, 3%).

All patients were treated with single or combination ICI; in particular, anti-PD1 were adopted in 19/35 (54%) (nivolumab *n* = 9, pembrolizumab *n* = 9, toripalimab *n* = 1), anti-PDL1 in 9/35 (26%) (atezolizumab *n* = 8, avelumab *n* = 1), CTLA4-inhibitor in 1/35 (3%) (ipilimumab *n* = 1). Combination therapy was administered to 6/35 (17%) patients and consisted in either anti-PD1 and CTLA4-inhibitor (nivolumab and ipilimumab *n* = 4), anti-PDL1 and CTLA4-inhibitor (durvalumab and ipilimumab *n* = 1), or anti-PD1 and anti-LAG3 (nivolumab and relatlimab *n* = 1). Nineteen patients also received chemotherapy and/or radiotherapy. Cerebellar toxicity developed a median of 11 weeks after the onset of ICI therapy (range: 0.1–82; data available for 33 patients). In 11/33 (33%) patients, toxicity arose after 6 months of ICI initiation.

Full-blown neurological symptoms consisted in “isolated cerebellar ataxia” (gait and/or limb and/or trunk ataxia) in 13/35 (37%), ataxia and dysarthria in 6/35 (17%), ataxia and ocular involvement in 4/35 (11%) and a “pancerebellar syndrome” (ataxia, dysarthria, and ocular involvement) in 9/35 (26%). Symptoms could not be further characterized in 3/35 (9%) patients.

Four patients developed other irAE, namely inflammatory arthritis, diabetic ketoacidosis due to type I diabetes and possible pancreatitis, hyponatremia and arthritis, as well as vitiligo and thyroiditis. Ab testing was not available in 4 patients; positivity was detected in 15/31 (48%) cases. Abs included those binding to unclassified antigens (*n* = 2), anti-Hu (*n* = 2), anti-Yo (*n* = 2), anti-Yo and anti-VGCC (*n* = 1), anti-PCA-2 (*n* = 1), anti-Zic4 (*n* = 1), anti-GAD65 (*n* = 1), anti-GFAP (*n* = 1), anti-TRIM9 (*n* = 1), neurofilament-light chain, neurofilament-heavy chain and alpha-internexin (*n* = 1), amphiphysin and P/Q VGCC (S) (*n* = 1), and anti-DACH1 (*n* = 1).

Lumbar puncture was not available in five patients. CSF findings included raised protein content (21/30, 70%) and pleocytosis (15/30, 50%). OCBs were tested in 14/30 (47%) patients and were present in all but 3 of them. Overall, CSF was inflammatory in 25/30 (83%). Search for malignant cells was negative in all cases.

Brain MRI was available for all patients; 8/35 (23%) showed cerebellar hyperintensities, 4/35 (11%) cerebellar atrophy, and 2/35 (6%) cerebellar edema. Additional findings included early tonsillar herniation and hydrocephalus (*n* = 1), brain small vessel disease (*n* = 1), multiple spot-shaped periventricular, leptomeningeal and parenchymal contrast enhancements (*n* = 1), leptomeningeal enhancement (*n* = 1), bilateral III and right VII and VIII cranial nerve enhancement (*n* = 1), cortical ribbon interruption (pre-existent) and supratentorial metastases (*n* = 1), T2/FLAIR hyperintensity of the left oculomotor nerve with contrast enhancement (*n* = 1).

**Fig. 2:**
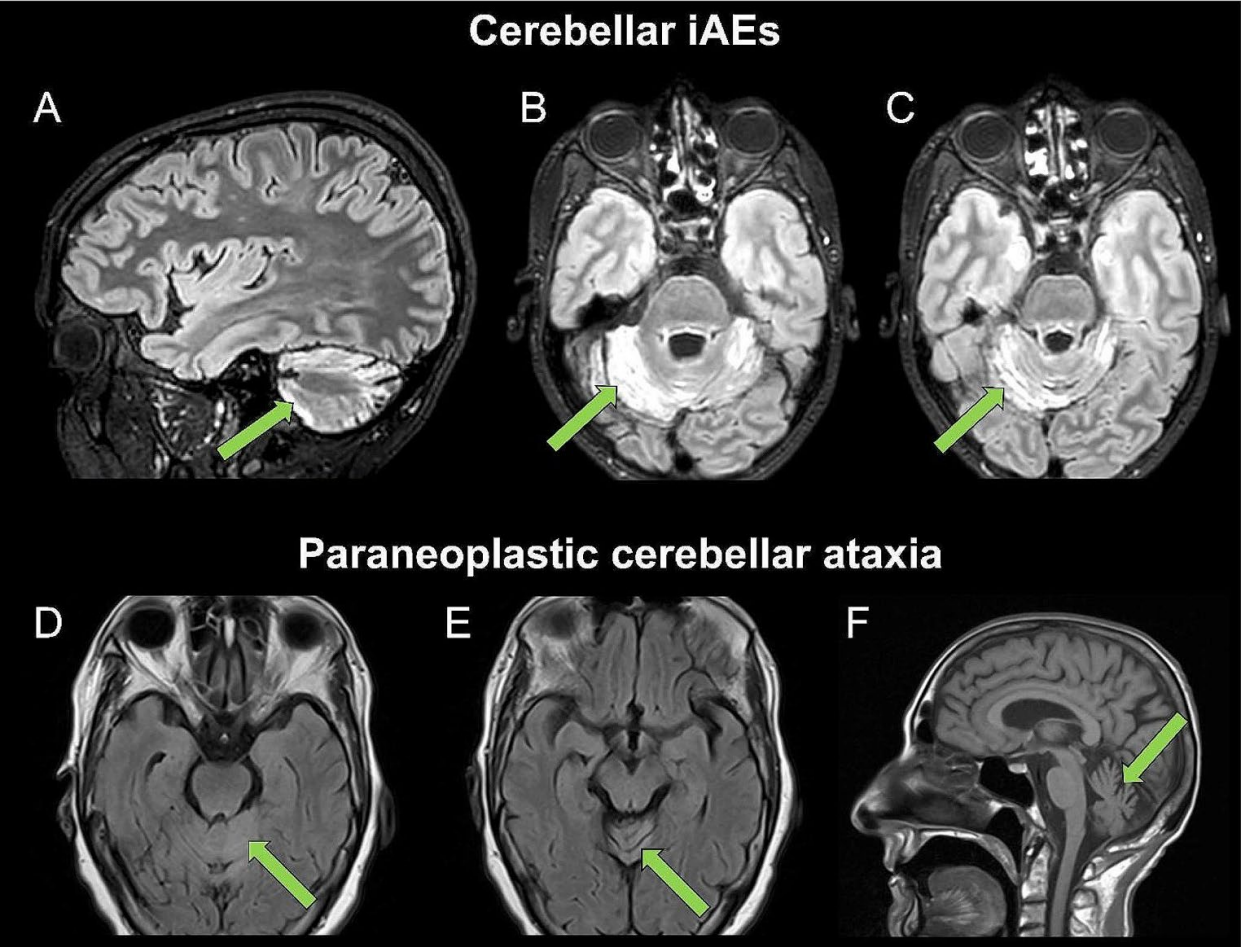
Representative brain MRI findings. Fluid attenuated inversion recovery (FLAIR) brain magnetic resonance imaging (MRI) findings in patients with cerebellar immune-related adverse events (irAEs) due to immune checkpoint inhibitors cancer treatment (**A**, sagittal section; **B** and **C**, axial sections) showing cerebellar hyperintensities. Brain MRI findings in paraneoplastic cerebella ataxia demonstrating mild cerebellar hypersignal (**D** and **E**, axial sections) as well as atrophy (**F**, sagittal section)

**Fig. 3:**
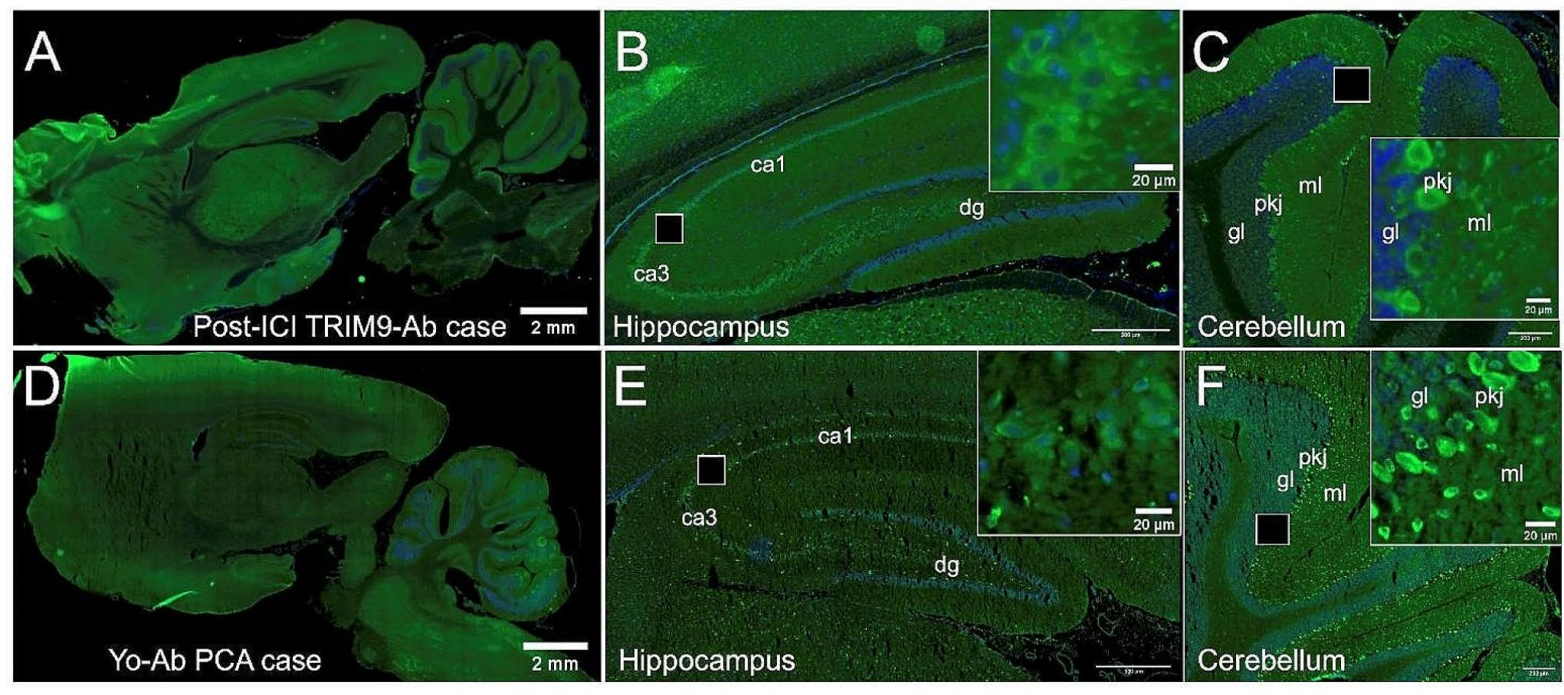
Immunological studies. Indirect fluorescent immunostaining on rat brain sections performed using Patient #5 cerebrospinal fluid (CSF) (**A**, **B** and **C**) or CSF from a patient with classical anti-Yo paraneoplastic cerebellar ataxia (PCA) (**D**, **E** and **F**). Patient #5 CSF showed the typical staining pattern of TRIM9-Abs: strong staining of CA3 pyramidal cells in the hippocampus (**B**), strong reactivity at the level of the molecular layer, the cell bodies, and proximal dendrites of Purkinje cell in the cerebellum (**C**). Classical Yo-Abs produce a strong staining of the cytoplasm of Purkinje cells. Basket and stellate cells in the molecular layer are also labeled (**F**). Ca1, ca3: CA1 and CA3 pyramidal cell layer of the hippocampus; dg: dentate gyrus; gl: granular layer, pkj: purkinje cell layer, ml: molecular layer of the cerebellum

Brain MRI findings of patients with cerebellar irAEs are shown in Fig. 2 and compared to those of patients with PCA, while results of indirect immunofluorescence are shown in Fig. 3.

ICI was withdrawn in all patients. Immune-modulating therapy was applied in 33/35 patients (94%). It consisted in CS monotherapy in 18/35 (51%), CS and IVIg in 7/35 (20%), CS and infliximab in 1/35 (3%), a combination of three among CS, IVIg, Plasma Exchange (PE) and RTX in 4/35 (11%), CS, IVIg and tofacitinib in 1/35 (3%), and CS, IVIg, PE and RTX in 1/35 (3%). Two patients received no immune-modulating therapy (2/35, 6%).

Concerning neurological outcome, 16/35 (46%) patients improved with residual disability, 9/35 (26%) returned to pre-ICI condition, 7/35 (20%) did not improve and 3/35 (9%) worsened. By applying the PNS-Care Score [14] to patients with cerebellar irAEs, “PNS-like” syndromes were identified. Three patients belonged to the “definite” score category (3/35, 9%), nineteen to the “probable” (19/35, 54%), and thirteen to either the “possible” or “non-PNS” (13/35, 37%). When comparing patients with “definite” PNS-like syndromes to the remainders, a statistically significant worse neurological outcome was identified (*p* < 0.05) (Supplementary Table 1).

Tumor regression or no progression was observed in 14/35 (40%), while 10/35 (29%) relapsed; oncological outcome was not specified in 11/35 (31%). At last available follow-up, 8/35 (23%) patients had died due to either tumor progression (*n* = 5) or pneumonia (*n* = 2); in one case (*n* = 1) cause of death was undetermined.

### Paraneoplastic Cerebellar Ataxias

We identified 15 cases of PCA at the Udine University Hospital during 7 years (2017–2023). Relevant characteristics of patients with PCAs are shown in Table 2.

**Table 2:** Characteristics of patients with paraneoplastic cerebellar ataxia

Patient	Sex, age	Underlying tumor	Time lag, onset of neurological symptoms-histological tumor diagnosis (weeks)	Neurological symptoms	Abs	MRI/CSF (increased protein content and pleocytosis)	Treatment	Neurological outcome
1	W, 59	Merkel-cell carcinoma	13	Ataxia + ocular	NfM	-/--	CS, IVIg, PE	1
2	W, 65	ovarian	39*	Pancerebellar	Yo	-/NA	CS, IVIg	1
3	W, 73	*unknown*	NA	Ataxia + dysarthria	Yo	-/--	CS	NA
4	M, 33	leukemia	70*	Ataxia + dysarthria	-	-/+-	none	1
5	W, 75	ovarian	87*	Ataxia + dysarthria	Yo	Cerebellar hyperintensities/NA	CS	1
6	M, 74	prostate	183	Ataxia + dysarthria	-	NA/NA	none	0
7	W, 73	ovarian	522*	Ataxia + ocular	-	-/--	none	2
8	M, 71	gastric	7	Ataxia + ocular	Ma2	-/--	CS, IVIg	2
9	W, 44	breast	15	Pancerebellar	Yo	Cerebellar atrophy/++	CS, IVIg, PE, cyclophosphamide	3
10	M, 71	NSCLC	46*	Ataxia + dysarthria	-	-/--	CS	NA
11	W, 77	*unknown*	NA	Pancerebellar	Yo	-/NA	CS, IVIg	2
12	W, 53	breast	8	Ataxia + ocular	Yo	-/NA	CS	2
13	W, 70	gastric§	81*	Isolated ataxia	-	Cerebellar atrophy/NA	none	2
14	W, 68	esophageal	0	Ataxia + ocular	-	NA/NA	none	2
15	M, 60	SCLC, prostate	0	Ataxia + dysarthria	Hu	Cerebellar atrophy/NA	CS, IVIg	2

*Abbreviations* Ab, antibody; CS, corticosteroids; CSF, cerebrospinal fluid; IVIg, intravenous immunoglobulins; M, man; MRI, magnetic resonance imaging; NA, not available; NfM, neurofilament medium chain; NSCLC, non-small cell lung cancer; PE, plasma exchange; SCLC, small cell lung cancer; W, woman; +, present; -, negative

Neurological outcome: 0 = return to baseline condition; 1 = improvement with residual disability; 2 = no improvement; 3 = worsening

*onset of neurological symptoms was posterior to tumor diagnosis

§ the patient also had a previous tumor (lung adenocarcinoma, diagnosed 8 months prior to gastric cancer)

Among PCA patients, 10/15 (67%) were females, while 5/15 (33%) were males. The median age at neurological disease onset was 70 years (age range: 33–77). The most frequent associated tumor was ovarian cancer (*n* = 3), followed by breast cancer (*n* = 2), gastric cancer (*n* = 2), NSCLC (*n* = 1), Merkel-cell carcinoma (*n* = 1), prostate cancer (*n* = 1), esophageal cancer (*n* = 1), leukemia (*n* = 1) and concomitant prostate and SCLC (*n* = 1). In 2 cases tumor status was unknown. One patient only had a previous cancer (lung adenocarcinoma, diagnosed 8 months prior to gastric cancer). Seven patients were metastatic at tumor diagnosis (7/15, 47%), while in 4/15 (27%) the tumor was localized; data were not available in 4/15 (27%).

Full-blown neurological symptoms consisted in ataxia and dysarthria in 6/15 (40%), ataxia and ocular involvement in 5/15 (33%), a “pancerebellar syndrome” (ataxia, dysarthria, and ocular involvement) in 3/15 (20%), and “isolated cerebellar ataxia” (gait and/or limb and/or trunk ataxia) in 1/15 (7%).

These symptoms developed a median of 39 weeks from histological tumor diagnosis (range: 0-522); symptoms were antecedent to tumor diagnosis in 7/15 (47%) cases, while in 8/15 (53%) they were detected simultaneously or after oncological diagnosis. Ab positivity was detected in 9/15 (60%) cases, namely anti-Yo (*n* = 6), anti-Ma2 (*n* = 1), anti-Hu (*n* = 1), and anti-neurofilament medium chain (*n* = 1). Lumbar puncture was performed in 7/15 cases (47%). Findings included raised CSF proteins (2/7, 29%) and pleocytosis (1/7, 14%). MRI was not available in 2/15 (13%) patients; 3/15 (20%) showed cerebellar atrophy, and 1/15 (7%) cerebellar hyperintensities; in 9/15 (60%) cases no cerebellar abnormality was detected. Additional findings included gliosis and/or meningioma (not related to the clinical picture) and/or hyperintense lesions in 7/15 cases (47%).

Immune-modulating therapy was applied in 10/15 patients (67%). It consisted in CS in single therapy in 4/15 (27%), CS and IVIg in 4/15 (27%), CS, IVIg and PE in 1/15 (7%), a combination of CS, IVIg, PE and cyclophosphamide in 1/15 (7%); no treatment was applied in 5/15 (33%). Concerning neurological outcome, 1/15 (7%) patients returned to baseline condition, 4/15 (27%) improved with residual disability, 7/15 (47%) did not improve and 1/15 (7%) worsened. Outcome was not available in 2/15 (13%) cases. When calculating the PNS-Care Score [14], 5/15 (33%) had a definite PNS, 5/15 (33%) a probable PNS, and 5/15 (33%) a possible PNS.

Oncological follow up was not available in 4/15 (27%) cases. Tumor regression or no progression was observed in 8/11 (73%), while 2/11 (18%) relapsed; in 1/11 (9%) a tumor was not found.

### Comparison between Cerebellar irAEs and Paraneoplastic Cerebellar Ataxias

A comparison between cerebellar irAEs and paraneoplastic cerebellar ataxias is presented in Table 3. Female patients were significantly more represented in the PCA group (*p* = 0.01). Ab positivity did not differ between the two group, while high-risk Abs were significantly more represented among PCA patients (*p* < 0.05), and so were anti-Yo Abs specifically (*p* < 0.05). Gynecological and breast cancers were significantly more frequent in the PCA group (*p* < 0.01), while lung cancer in the cerebellar irAE group (*p* < 0.01). As far as concerns the clinical presentation, “isolated cerebellar ataxia” was more common in the cerebellar irAE group (*p* < 0.05), while ataxia associated with either dysarthria *or* ocular symptoms was more frequent in the paraneoplastic group (*p* < 0.01). In addition, patients who developed cerebellar toxicity following ICI showed a significantly better neurological outcome (*p* < 0.05) (a good neurological outcome being defined as neurological improvement or return to baseline condition). Immune-modulating therapy was more frequently applied in the cerebellar irAEs group as compared to the PCA group (*p* < 0.05).

**Table 3:** Comparison between cerebellar irAE and PCA

Features	Cerebellar irAE	PCA	*p* Value
Sex, n (%)	*N* = 35	*N* = 15	
Female	10 (29)	10 (67)	**0.01**
Male	25 (71)	5 (33)	
Abs, n (%)	*N* = 31	*N* = 15	
Ab positivity	15 (48)	9 (60)	> 0.05
*High risk Abs*	*7*	*8*	***< 0.05***
*[Anti-Yo]*	[3]	[6]	***[< 0.05]***
*[Anti-Hu]*	[2]	[1]	*[> 0.05]*
*UNCA*	*2*	*0*	*> 0.05*
*Other Abs*	*6*	*1*	*> 0.05*
Ab negativity	16 (52)	6 (40)	
Tumor type, n (%)	*N* = 35	*N* = 15	
NSCLC	12 (34)	1 (7)	> 0.05
SCLC	9 (26)	1 (7)	> 0.05
Lung cancer (NSCLC and SCLC)	21 (60)	2 (13)	**< 0.01**
Gynecological and breast cancer	1 (3)	5 (42)	**< 0.01**
Symptoms, n (%)	*N* = 32	*N* = 15	
“isolated cerebellar ataxia”	13 (37)	1 (7)	**< 0.05**
ataxia and dysarthria	6 (17)	6 (40)	> 0.05
ataxia and ocular involvement	4 (11)	5 (33)	> 0.05
ataxia and (ocular involvement *or* dysarthria)	10 (28)	11 (73)	**< 0.01**
“pancerebellar syndrome”	9 (26)	3 (20)	> 0.05
Immune-modulating therapy, n (%)	*N* = 35	*N* = 15	
Yes	33 (94)	10 (67)	**< 0.05**
None	2 (6)	5 (33)	
Neurological outcome, n (%)	*N* = 35	*N* = 13	
Improvement	25 (71)	5 (33)	**< 0.05**
No improvement/worsening	10 (29)	8 (53)	

*Abbreviations* Ab, antibody; irAE, immune-related adverse event; n, number; N, total number of patients considered as per data availability; NSCLC, non-small cell lung cancer; PCA, paraneoplastic cerebellar ataxia; SCLC, small cell lung cancer; UNCA, antibodies binding to unclassified antigens

**Fig. 4:**
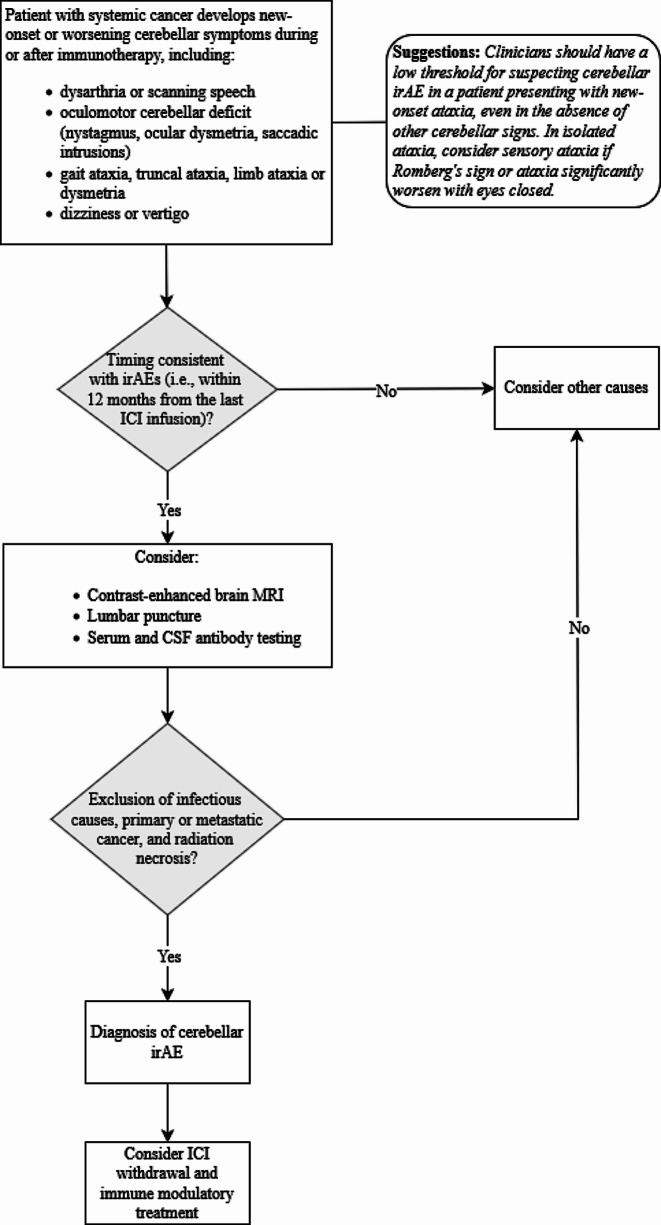
Diagnostic algorithm specific to cerebellar irAEs

## Discussion

In the present study, we characterized cerebellar toxicity following ICI administration and compared it to its paraneoplastic counterpart (PCA). We observed several differences between these two disorders:

(i) cerebellar irAEs tend to be associated with male sex as compared to the classic female predominance of PCA. Male predominance in the cerebellar irAE group may be justified by cancer prevalence and epidemiology, as well as sex-based differences in ICI use. Gender-based differences in tumor response may as well account for dissimilarity in the prevalence of irAEs [15]. Interestingly, previous studies focused on neurological irAEs have shown a similar male predominance (approximately 70% vs. 30%) of complications affecting the CNS [7, 16].
(ii) lung cancer (in particular NSCLC) is the typical malignancy observed in cerebellar irAEs, while gynecological tumors and breast cancer are more frequent in PCA, especially in anti-Yo PCA. Interestingly, clinical presentation-Ab-tumor triad in cerebellar irAEs only partially reflects the typical associations of paraneoplastic disorders (i.e., three patients tested positive for anti-Yo Abs, but only one of them was a woman with ovarian cancer; the remainder were both males and affected by lung cancer). This may be because gynecological malignancies (typically associated with PCA) are not usually treated with ICIs, which are frequently adopted against lung cancer instead. This phenomenon has already been observed in other studies [17, 18]; interestingly, treatment with ICIs may shape cancer immunogenicity, modifying and increasing neoantigen expression while targeting the tumour [19]. The increasing use of ICIs may therefore uncover new onconeural antigens which may or may not be clinically relevant, leading to an overt disease or merely representing an epiphenomenon [17, 18]. When onconeural Abs are detected in a patient presenting with a compatible clinical phenotype (e.g. focal limbic encephalitis and anti-Ma2 or anti-Hu Abs), the Ab positivity against such intracellular epitopes usually harbors a poor prognosis [7, 20, 21].
(iii) cerebellar irAEs develop a median of 11 weeks after ICI initiation in patients with advanced-stage cancer, while in a group of PCA patients the neurological syndrome antedates the discovery of the tumor. The typical timeframe of neurological irAEs corresponds to the first 6 months of ICI onset, although they can still be defined as such if appearing within 12 months from the last ICI infusion [5]. Importantly, CNS complications tend to appear later than those affecting the peripheral nervous system (i.e. myositis and myasthenia gravis, and overlap syndromes) [22]. In our study, toxicity consistently developed while immunotherapy was ongoing; in about one third of the cases, it arose after 6 months of ICI initiation. Instead, one patient detected from the literature review developed hyperacute toxicity, with neurological symptoms beginning the day after toripalimab administration (Zhou et al. [23]). Though infrequent, hyperacute toxicities have been reported in the literature for both neurological and non-neurological irAEs [24–28]. Interestingly, the patient described by Zhou et al. tested positive for GAD65 Abs, which have been associated to cerebellar ataxia [14]; this finding suggests a possible predisposition to autoimmunity, enhanced by ICI exposure, although no samples taken before ICI introduction was tested for GAD65 Abs. An hyperacute course mimicking cerebrovascular disorders has been reported also for PCA in less than 10% of the cases [29].
(iv) the Ab profile of the cerebellar irAE group is quite heterogeneous, while well-characterized, onconeural (high-risk) Abs (especially anti-Yo) prevail in the PCA group. In the cerebellar irAE cohort, patients demonstrated Ab positivity to uncharacterized antigens, high risk Abs, and Abs which have shown association to cerebellar ataxia, though remaining less characterized (anti-TRIM9, neurofilament-light chain, and anti-Zic4 [30]). Neuronal antibodies were equally tested in the cerebellar irAEs and PCA group (Supplementary Table 2).
(v) the most common clinical presentation of cerebellar irAEs corresponds to “isolated cerebellar ataxia”, namely gait and/or trunk and/or limb ataxia, while ataxia with either ocular involvement or dysarthria is more frequent among PCA cases. Clinicians should therefore have a low threshold for suspecting ICI toxicity in a patient presenting with even mild, new-onset, isolated ataxia, despite the absence of other cerebellar signs.
(vi) From a diagnostic standpoint, CSF is more informative than MRI in the diagnosis of cerebellar irAE, as it was inflammatory in most patients, while MRI did not show any cerebellar abnormality in over half of them. This finding further confirms that MRI is unremarkable in a variable but consistent number of cases, as already demonstrated by several series on cerebellar and other CNS irAEs [6, 8, 16, 17]. Among pathological findings, cerebellar hyperintensities were the most common. Lumbar puncture should therefore be considered in all patients with suspected CNS irAEs for both confirming the diagnosis as well as to excluded potential mimics (e.g. infectious disorders and leptomeningeal carcinomatosis) [22]. A diagnostic algorithm specific to cerebellar irAEs is presented in Fig. 4.
(vii) The majority of patients developing cerebellar toxicity after ICI exposure was treated with CS, either alone or in combination, and overall, response to immune-modulating therapy appears to be quite good, especially if compared to PCA. This is in keeping with available data on paraneoplastic neurological syndromes, which tend to stabilize with appropriate oncological treatment, and rather show limited response to immune-modulating therapy, especially in the presence of high-risk neuronal Abs [31]. In addition, the overrepresentation of anti-Yo PCA may account for the more severe presentation of the paraneoplastic group. However, it must also be taken into account that treatment was more frequently applied in the cerebellar irAEs group as compared to the PCA group.

Before this study, only small case series and case reports had described cerebellar irAEs, which were nicely put together in a recent review by Dinoto et al. [8]. As compared to this review, our series appears to be more restrictive, as we included only patients presenting with de-novo isolated or predominant cerebellar dysfunction, while patients with pre-existent cerebellar conditions or those with multifocal involvement were here excluded in order to have a clear clinical characterization of this condition. Despite more stringent inclusion criteria, our cerebellar irAE cohort appears more numerous (*n* = 35 vs. *n* = 15 isolated cerebellitis in the study by Dinoto et al.) thanks to the inclusion of original cases. Similarly to Dinoto et al. [8], our study found that lung cancer, unremarkable brain MRI, inflammatory CSF, and favorable response to immune-modulating therapy tend to be associated with cerebellar irAEs.

The present study is limited by its retrospective nature, small sample size, heterogenicity of oncological and neurological follow-up and, possibly, referral bias toward more complex cases. Nevertheless, it represents the 7-year experience of three centers (including a national reference center) focused on the diagnosis and treatment of neurological irAEs and PNS.

## Conclusion

In conclusion, we characterized cerebellar toxicity following ICI administration, providing a description of timing of its occurrence, tumor association, clinical and paraclinical features, and outcome. Interestingly, clinical presentation-Ab-tumor triad in the ICI group only partially reflects the typical associations of paraneoplastic disorders. To our knowledge, this represents the largest cohort of new onset, isolated or predominant cerebellar toxicity following ICI administration and the only study comparing cerebellar irAEs to the naturally occurring PCA.

## Electronic Supplementary Material

Below is the link to the electronic supplementary material.

Supplementary Material 1

Supplementary Material 2

## Data Availability

Data is provided within the manuscript or supplementary information files. Anonymized data not published within this article will be made available upon reasonable request from any qualified investigator.
