# Supplementary material for: Immune Checkpoint Inhibitor-Related Cerebellar Toxicity: Clinical Features and Comparison with Paraneoplastic Cerebellar Ataxia
Source: Cerebellum. 2024 Aug 17;23(6):2308–23. doi: 10.1007/s12311-024-01727-5 (PMC11585521; doi:10.1007/s12311-024-01727-5)
Supplement: Supplementary file 2 — Supplementary Material 2 [file 12311_2024_1727_MOESM2_ESM.docx]

**Supplementary Table 1: PNS-Care Score applied to patients with cerebellar irAEs**

| Features | Good neurological outcome (0-1) | Poor neurological outcome (2-3) | *p* Value |
| --- | --- | --- | --- |
| PNS-Care Score, n (%) | **N=25** | **N=10** |  |
| Definite | 0 (0) | 3 (30) | **p<0.05** |
| Others (probable *or* possible *or* non-PNS) | 25 (100) | 7 (70) |  |

*Abbreviations: n, number; N, total number of patients; PNS, paraneoplastic syndrome.*

*Neurological outcome: 0 = return to pre-ICI condition; 1= improved with residual disability; 2 = no improvement; 3 = worsening*

**Supplementary Table 2: Neuronal Ab testing in patient with cerebellar irAEs and PCA**

| Features | Cerebellar irAEs | PCA | *p* Value |
| --- | --- | --- | --- |
| Ab testing, n (%) | **N=35** | **N=15** |  |
| Number of patients tested | 31 (89) | 15 (100) | p>0.05 |
| Number of patients not tested | 4 (11) | 0 (0) |  |

*Abbreviations: Ab, antibody; irAE, immune-related adverse event; n, number; N, total number of patients; PCA, paraneoplastic cerebellar ataxia*
